# Factors Associated With Death at 30 Days and Evaluation of Clinical Risk Scores Among Patients With Cancer Admitted With Postchemotherapy Infection in Uganda: A Prospective Cohort Study

**DOI:** 10.1093/ofid/ofae634

**Published:** 2024-10-25

**Authors:** Ambaru Jacinta Ojia, Sophie E Lyon, Jane Francis Birungi, Catherine Owomugisha, Rose Muhindo, Semei Buwambaza Sekitene, Christopher C Moore, Edwin Nuwagira

**Affiliations:** Department of Medicine, Mbarara University of Science and Technology, Mbarara, Uganda; Department of Oncology, Uganda Cancer Institute, Kampala, Uganda; Division of Infectious Diseases and International Health, University of Virginia, Charlottesville, Virginia, USA; Oncology Unit, Mbarara Regional Referral Hospital, Mbarara, Uganda; Oncology Unit, Mbarara Regional Referral Hospital, Mbarara, Uganda; Department of Medicine, Mbarara University of Science and Technology, Mbarara, Uganda; Department of Medicine, Mbarara University of Science and Technology, Mbarara, Uganda; Department of Oncology, Uganda Cancer Institute, Kampala, Uganda; Department of Medicine, Mbarara University of Science and Technology, Mbarara, Uganda; Division of Infectious Diseases and International Health, University of Virginia, Charlottesville, Virginia, USA; Department of Medicine, Mbarara University of Science and Technology, Mbarara, Uganda

**Keywords:** cancer, infection, mortality risk scores, sepsis, Uganda

## Abstract

**Background:**

Little is known about outcomes from cancer chemotherapy–-associated infections in sub-Saharan Africa. Accordingly, among patients with cancer admitted with postchemotherapy infection in Mbarara, Uganda, we aimed to determine (1) the 30-day case fatality rate, (2) factors associated with mortality rate, and (3) clinical risk score performance.

**Methods:**

We enrolled participants aged ≥18 years if they (1) received cancer chemotherapy within the past 30 days, (2) were admitted to the oncology ward, and (3) were prescribed intravenous antibiotics. We used Cox proportional hazards regression to determine predictors of death at 30 days and calculated the area under the receiver operating characteristic curve (AUC) for each clinical risk score.

**Results:**

Among 150 participants, 67 (45%) were female, and the median (interquartile range) age was 56 (43–66) years. Esophageal cancer (18%) and pneumonia (42%) were the most common cancer and infection, respectively. Death occurred within 30 days in 63 participants (42%). Quick Sequential Organ Failure Assessment (qSOFA) score ≥2 (adjusted hazard ratio, 2.51 [95% confidence interval, 1.42–4.44]; *P* = .001), and Universal Vital Assessment (UVA) score >4 (2.13 [.08–4.18, *P* = .03) were independently associated with death at 30 days. An Eastern Cooperative Oncology Group (ECOG) score ≥3 was similarly independently associated with death at 30 days in the qSOFA and UVA models. The AUCs for qSOFA and UVA scores were 0.70 (95% confidence interval, .63–.79) and 0.72 (.64–.80), respectively.

**Conclusions:**

In participants with postchemotherapy infection in Mbarara, Uganda, the case fatality rate was high. ECOG, qSOFA, and UVA scores were associated with death at 30 days.

Cancer is a leading cause of death worldwide, and the number of new annual cases is expected to rise from 19.3 million in 2020 to 30.2 million by 2040 [1]. Almost 70% of all cancer deaths occur in low- and middle-income countries (LMICs) [2, 3]. Cancer chemotherapy is increasingly available in LMICs; however, as the prevalence of cancer rises globally, this disproportionate mortality burden is expected to continue to rest on LMICs [3].

Although cancer treatment improves survival rates, the cytotoxic effects of chemotherapy increase the risk of infection, which may lead to sepsis and death. Sepsis is the leading cause of global death and has a heightened morbidity and mortality risk among patients with cancer receiving chemotherapy [4, 5]. This vulnerability is particularly pronounced in patients living in LMICs, including those living in sub-Saharan Africa (sSA) [4, 6]. In sSA, human immunodeficiency virus (HIV), undernutrition, tuberculosis, malaria, antimicrobial resistance, and resource limitations contribute to infection-related disease and deaths [7]. At the national referral Uganda Cancer Institute in Kampala, the 30-day case fatality rate for patients with solid and liquid tumors that were complicated by postchemotherapy infection was 25% and 46%, respectively [6, 8]. However, data are lacking regarding mortality rate for postchemotherapy infection from regional cancer treatment centers in Africa.

The definition of sepsis has evolved from systemic inflammatory response syndrome based criteria to include organ failure, which is operationally defined as a ≥2-point increase in the Sequential Organ Failure Assessment (SOFA) score in the setting of infection [9]. To identify patients at increased mortality risk outside an intensive care unit, a quick SOFA (qSOFA) score was derived [9]. More recently, the Universal Vital Assessment (UVA) score was derived and validated as a mortality risk score for use in hospitalized patients in sSA [10, 11]. However, there are no data regarding the ability of clinical risk scores to predict mortality rates in patients with cancer in sSA with infection after recent chemotherapy. The objectives of the current study were to determine (1) the 30-day case fatality rate, (2) factors associated with mortality rate, and (3) qSOFA and UVA risk score performance among patients with cancer admitted to Mbarara Regional Referral Hospital (MRRH) with postchemotherapy infection.

## METHODS

### Study Setting

This study was conducted at the oncology ward of MRRH, the teaching hospital for the Mbarara University of Science and Technology located in Mbarara, Uganda. The oncology ward at MRRH was established in 2013 as a satellite unit of the Uganda Cancer Institute (Kampala) and was one of 2 public cancer treatment facilities in Uganda at the time of the study. The MRRH oncology ward provides inpatient and outpatient care for adults and registers an average of 30 new patients per month. Outpatient clinics occur twice weekly, during which patients receive oncology care, including chemotherapy infusions. Approximately 40 patients attend the oncology clinic each day, and the oncology ward includes 20 beds. Since its inception, >5000 patients have been registered in the cancer treatment program at MRRH.

### Study Design and Data Collection

We conducted an analysis of a prospective cohort of participants who were enrolled in the study from 4 July 2022 through 9 February 2023. We applied Slovin's formula to determine the sample size needed to estimate the case fatality rate following chemotherapy-induced infections. Based on a 95% confidence interval (CI) and a 5% margin of error, and assuming a conservative 15% case fatality rate for these infections from comparable studies, we calculated that ≥128 participants would be necessary. We enrolled participants aged ≥18 years if they (1) received cancer chemotherapy within the past 30 days, (2) were admitted to the oncology ward, and (3) were prescribed intravenous antibiotics. As blood cultures are not routinely available at MRRH, we defined suspected infection as documented administration of intravenous antibiotics at the time of admission. We excluded patients if they received chemotherapy within 24 hours of admission to hospital or if their clinical and laboratory data were collected >24 hours from the time of admission.

The admitting medical team provided clinical care independently from the study team. All study participants underwent an interview with the study team, which also conducted a chart review to collect clinical data—demographic information, oncology history, and infection history, including HIV serostatus, tuberculosis history, and postchemotherapy infection. We determined the Eastern Cooperative Oncology Group (ECOG) performance status scale score for each participant. The study team obtained vital signs from each participant at the time of admission to hospital. Acquisition of laboratory tests was not included in the study protocol, so we collected available laboratory data from the participant's clinic chart. We recorded the date of discharge or death for each participant. We contacted discharged participants or their next of kin at 30 days after enrollment to determine their vital status.

### Statistical Analysis

We recorded data in a Castor electronic database (version 2023.2.2.1) and performed analyses using SPSS software (version 29.0.2.0 [20]). We summarized categorical participant characteristics as frequency with percentage and continuous characteristics as median with interquartile range. We used the χ^2^ test to compare categorical variables. To compare continuous variables, we first assessed the normality of the data distribution using Kolmogorov-Smirnov and Shapiro-Wilk tests. For data with a normal distribution, we applied an independent *t* test, and for nonnormally distributed data, we used the Mann-Whitney *U* test. We constructed Cox proportional hazards regression models to predict death at 30 days. We adjusted the multivariable models for age and sex and made an a priori decision as to which covariates to include in the models based on known mortality risk factors from the medical literature and our primary objective of evaluating clinical risk scores [12]. Due to statistical interaction, we created separate models that included qSOFA and UVA scores. We considered a 2-sided *P* value <.05 to imply statistical significance.

We calculated the qSOFA and UVA risk scores for each participant using clinical data obtained at the time of admission to the oncology ward (Supplementary Table 1). To determine discriminative ability for death at 30 days, we calculated the area under the receiver operating characteristic curve (AUC) for each clinical risk score. We compared AUCs using the DeLong test. We determined the sensitivity, specificity, positive predictive value (PPV), and negative predictive value for death at 30 days according to (1) clinician-diagnosed sepsis; (2) a threshold of ≥2 for the qSOFA score; and (3) <2 (low risk), 2–4 (medium risk), and >4 (high risk) for the UVA score. Participants with missing vital signs from hospital admission and those lost to follow-up were excluded from the analysis.

### Ethics Statement

This study was conducted in accordance with the ethical principles stated in the Declaration of Helsinki (1996) and the Uganda National Council for Science and Technology National Guidelines of Research Involving Humans as Research Participants (UNCST-HS3111ES). We obtained written approval to conduct the study from the Mbarara University of Science and Technology (REC-2022–452). All participants provided written informed consent before enrollment in the study. If a participant could not provide consent, then an accompanying next of kin, a family member or friend, provided it for them.

## RESULTS

### Patient Characteristics

We identified 164 patients who met the study inclusion criteria (Supplementary Figure 1). Of these, 154 participants (94%) were enrolled, of whom 4 (3%) were lost to follow-up. Among the 150 participants available for analysis, 67 (45%) were female, and the median age (interquartile range) was 56 (43–66) years (Table 1). The most common cancer was esophageal cancer (18%), followed by gastric cancer (12%) and breast cancer (10%) (Supplementary Table 2). There were 45 participants (30%) with an in-dwelling medical device. The most common clinical diagnoses were pneumonia (42%), sepsis (36%), and gastroenteritis (14%) (Supplementary Table 3). Of the 139 participants with known HIV status, 20 (14%) were living with HIV, of whom 5 (25%) had a history of tuberculosis, compared to none of the 119 participants without HIV (*P* < .001).

**Table 1.** Demographic and Clinical Data in Patients With Cancer Admitted With Postchemotherapy Infection to Mbarara Regional Referral Hospital, Uganda, 2022–2023

Variable	Median Value (IQR)^a^			*P* Value
	All Patients (n = 150)	Patients Who Survived (n = 87)	Patients Who Died (n = 63)	
Female sex, no. (%)	67 (45)	37 (43)	30 (48)	.54
Age, years	56 (43–66)	55 (42–67)	58 (43–66)	.59
Temperature, ^o^C	36.6 (35.9–37.7)	36.8 (36.3–37.8)	36.0 (35.6–37.7)	.07
Pulse rate, no./min	105 (90–118)	101 (91–117)	110 (87–119)	.64
Respirations, no./min	24 (20–32)	24 (20–30)	25 (20–32)	.44
SBP, mm Hg	102 (90–114)	107 (98–119)	91 (84–106)	<.001
Oxygen saturation, %	96 (92–98)	96 (95–98)	94 (90–96)	<.001
GCS score <15, no. (%)	36 (24)	8 (9)	28 (44)	<.001
WBC count, ×10^3^/µL	6.3 (3.1–10.0)	6.8 (3.1–11.8)	5.5 (3.2–9.1)	.34
ANC, ×10^3^/µL	3.5 (1.3–6.7)	3.8 (1.2–7.3)	3.3 (1.3–6.3)	.72
Hemoglobin, g/dL	10.0 (7.2–12.5)	10.0 (7.2–12.4)	9.8 (7.2–12.7)	.86
Creatinine, mg/dL	0.86 (0.67–1.24)	0.83 (0.64–1.14)	0.96 (0.68–1.48)	.10
Indwelling medical device, no. (%)	45 (30)	18 (21)	27 (43)	.004
ECOG score	2 (2–3)	2 (2–2)	3 (2–3)	<.001
Chemotherapy cycle no.	2 (1–4)	2 (1–4)	3 (1–4)	.71
Living with HIV, no. (%)	20 (14)	8 (10)	12 (21)	.07
Hematologic cancer, no. (%)	21 (14)	16 (18)	5 (8)	.07
Clinician-diagnosed sepsis, no. (%)	54 (36)	25 (29)	29 (46)	.03
Pneumonia, no. (%)	66 (44)	40 (46)	26 (41)	.33
Score category, no. (%)				
qSOFA ≥2	60 (40)	22 (25)	38 (60)	<.001
UVA <2 ( low risk)	54 (27)	40 (46)	14 (22)	.003
UVA 2–4 (medium risk)	60 (40)	37 (43)	23 (37)	.46
UVA >4 (high risk)	36 (24)	10 (11)	26 (41)	<.001

Abbreviations: ANC, absolute neutrophil count; ECOG, Eastern Cooperative Oncology Group; GCS, Glasgow Coma Scale; HIV, human immunodeficiency virus; IQR, interquartile range; qSOFA, quick Sequential Organ Failure Assessment; SBP, systolic blood pressure; UVA, Universal Vital Assessment; WBC, white blood cell.

^a^Values represent median (IQR) values where otherwise specified. Values were missing in ≤5% except for creatinine (missing in 24 [16%]) and HIV serostatus (missing in 11 [7%]).

### Death at 30 Days

Death occurred within 30 days in 63 participants (42%); 41 deaths (65%) occurred in the hospital. In the univariate analysis, systolic blood pressure ≤90 mm Hg, oxygen saturation <90%, Glasgow Coma Scale score <15, the presence of an indwelling medical device, ECOG score ≥3, qSOFA score ≥2, and UVA score >4 were associated with increased death at 30 days (Table 2). Hematologic cancer and UVA score <2 were associated with decreased death at 30 days. In the multivariable analysis, qSOFA score ≥2 (adjusted hazard ratio [aHR], 2.51 [95% CI, 1.42–4.44]; *P* = .001) and UVA score >4 (2.13 [1.08–4.18]; *P* = .03) were independently associated with increased death at 30 days (Table 3). An ECOG score ≥3 was similarly independently associated with increased death at 30 days in both the qSOFA (aHR, 1.92 [95% CI, 1.06–3.47]; *P* = .03) and UVA (2.32 [1.29–4.14]; *P* = .005) models (Table 3).

**Table 2.** Univariate Cox Proportional Hazards Regression Analysis of Clinical Predictors of death at 30 days in Patients With Cancer Admitted With Postchemotherapy Infection to Mbarara Regional Referral Hospital, Uganda, 2022–2023

Variable	HR	95% CI	*P* Value
Female sex	1.28	.78–2.10	.33
Age (y)	1.01	.99–1.02	.46
Temperature <36°C or >38°C	1.55	.94–2.54	.08
Heart rate >90/min	0.97	.56–4.70	.93
Respiratory rate >20/min	0.87	.51–4.48	.60
SBP ≤90 mm Hg	2.70	1.62–4.49	<.001
SpO_2_ <90%	1.84	1.03–3.30	.04
GCS score <15	2.45	1.49–4.02	<.001
WBC count <4×10^3^/µL or >12×10^3^/µL	0.88	.54–1.45	.62
ANC <1000/µL	0.69	.36–1.34	.27
Indwelling medical device	2.07	1.25–3.41	.004
ECOG score ≥3	2.88	1.65–5.01	<.001
Chemotherapy cycle no.	1.01	.92–1.11	.84
Hematologic cancer	0.37	.15–0.92	.03
Living with HIV	1.28	.68–2.41	.44
Pneumonia	0.93	.57–1.55	.79
Clinician-diagnosed sepsis	1.39	.85–2.28	.20
qSOFA score ≥2	2.75	1.65–4.58	<.001
UVA score <2 ( low risk)	0.49	.27–.89	.02
UVA score 2–4 (medium risk)	0.85	.51–1.42	.54
UVA score >4 (high risk)	2.37	1.43–3.91	<.001

Abbreviations: ANC, absolute neutrophil concentration; CI, confidence interval; ECOG, Eastern Cooperative Oncology Group; GCS, Glasgow Coma Scale; HIV, human immunodeficiency virus; HR, hazard ratio; qSOFA, quick Sequential Organ Failure Assessment; SBP, systolic blood pressure; SpO_2_, oxygen saturation; UVA, Universal Vital Assessment; WBC, white blood cell concentration.

**Table 3.** Multivariable Cox Proportional Hazards Regression Analysis of Clinical Predictors of death at 30 days, Adjusted for Age and Sex, in Patients With Cancer Admitted With Postchemotherapy Infection to Mbarara Regional Referral Hospital, Uganda, 2022–2023

Variable	aHR	95% CI	*P* Value
qSOFA score model			
ANC <1000/µL	1.08	.53–2.19	.84
Indwelling medical device	1.97	1.11–3.50	.02
ECOG score ≥3	1.92	1.06–3.47	.03
Hematologic cancer	0.32	.12–.84	.02
qSOFA score ≥2	2.51	1.42–4.44	.001
UVA score model			
ANC <1000/µL	0.94	.46–1.89	.86
Indwelling medical device	1.70	.91–3.16	.10
ECOG score ≥3	2.32	1.29–4.14	.005
Hematologic cancer	0.39	.15–1.05	.06
UVA score 2–4 (medium risk)	1.21	.60–2.43	.59
UVA score >4 (high risk)	2.13	1.08–4.18	.03

Abbreviations: aHR, adjusted hazard ratio; ANC, absolute neutrophil concentration; CI, confidence interval; ECOG, Eastern Cooperative Oncology Group; qSOFA, quick Sequential Organ Failure Assessment; UVA, Universal Vital Assessment.

### Evaluation of Clinical Risk Scores

**Figure 1.**
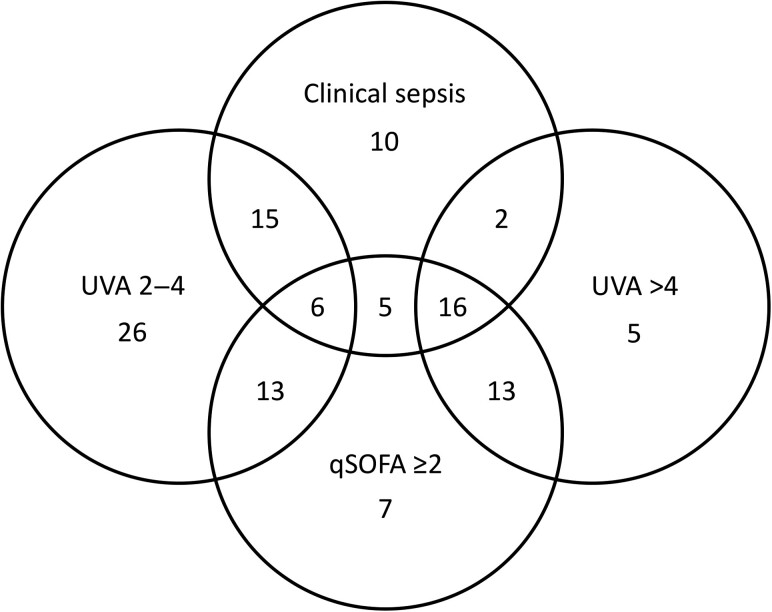
Venn diagram showing the overlap of sepsis diagnoses according to clinical diagnosis, quick Sequential Organ Failure Assessment (qSOFA) score ≥2, medium-risk Universal Vital Assessment (UVA) score (2–4), and high-risk UVA score (>4) among patients with cancer admitted with postchemotherapy infection at Mbarara Regional Referral Hospital, Uganda, 2022–2023.

The prevalences of clinician-diagnosed sepsis, qSOFA score ≥2, UVA score 2–4, and UVA score >4 were 54%, 40%, 40%, and 24%, respectively, with considerable overlap among definitions (Figure 1). Of the 54 participants with clinician-diagnosed sepsis, 27 (50%) met ≥2 qSOFA score criteria, compared with 33 (34%) of 96 without clinician-diagnosed sepsis (odds ratio, 1.91 [95% CI, .97–3.77]; *P* = .06). There were 18 participants (33%) with clinician-diagnosed sepsis who had a high-risk UVA score >4, compared with 18 (19%) of 96 without clinician-diagnosed sepsis (odds ratio, 2.2 [95% CI, 1.01–4.65]; *P* = .04).

**Figure 2.**
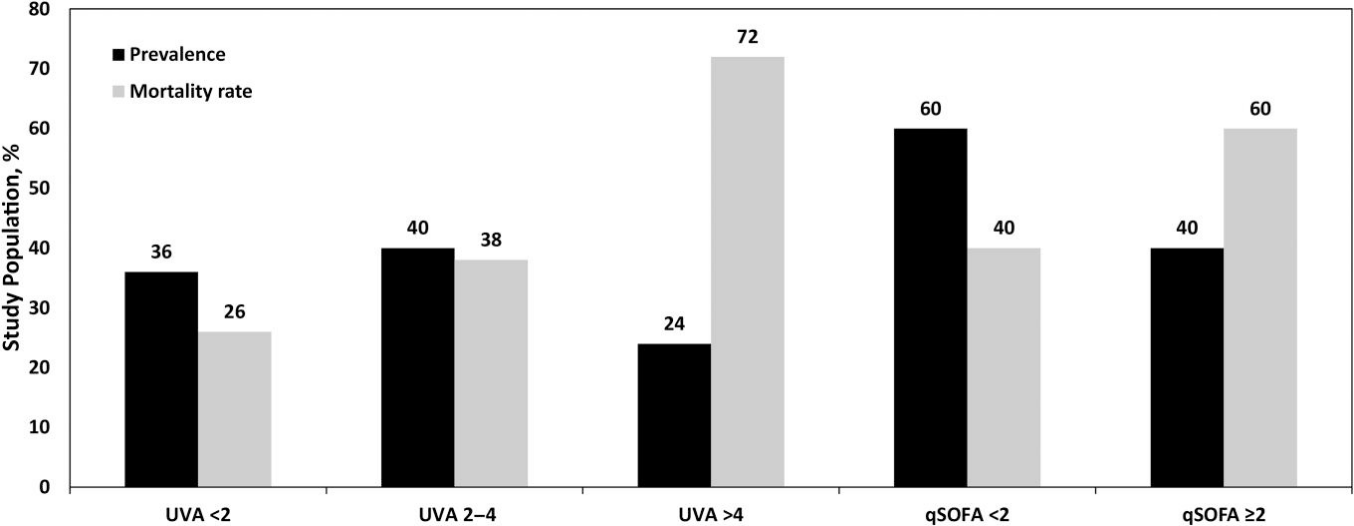
Prevalence and associated case fatality rates of Universal Vital Assessment (UVA) (<2, low-risk; 2–4, medium-risk; >4 high-risk) and quick Sequential Organ Failure Assessment (qSOFA; <2, low-risk; ≥2, high-risk) score categories among patients with cancer admitted with postchemotherapy infection at Mbarara Regional Referral Hospital, Uganda, 2022–2023.

Increasing qSOFA and UVA scores were associated with increased death at 30 days rate (Figure 2). The AUCs for qSOFA and UVA scores for discriminating death at 30 days were 0.70 (95% CI, .63–.79) and 0.72 (.64–.80), respectively, which did not differ significantly (DeLong comparison, *P* = .77). The PPVs (95% CI) for death at 30 days for qSOFA score ≥2 and UVA score >4 were 0.63 (.53–.72) and 0.72 (.58–.83), respectively, and did not differ significantly (standard error, 0.07 [95% CI, −.05 to .22]; *P* = .20) (Supplementary Table 4). In comparison, the PPV (95% CI) for death at 30 days for clinician-diagnosed sepsis was 0.46 (.36–.57).

## DISCUSSION

In this study of patients with cancer presenting with postchemotherapy infection in southwestern Uganda, nearly half of all participants died within 30 days of admission. Esophageal cancer was the most common underlying cancer. Microbiological diagnoses were not available, but pneumonia and sepsis were the most common clinician-diagnosed infections. There were few participants living with HIV, but a history of tuberculosis was common in those who were. Depending on the definition of sepsis used, the prevalence of sepsis was approximately 25%–50%. Clinician-diagnosed sepsis had the lowest PPV, and UVA score >4 had the highest PPV for death at 30 days. Independent factors associated with death at 30 days included high ECOG, qSOFA, and UVA scores. Both qSOFA and UVA scores had good discriminative ability for death at 30 days.

Lower respiratory tract infection is common in sSA and often leads to sepsis and acute respiratory distress syndrome [4, 13, 14]. In a previous study of adults hospitalized in sSA, our group found that severe respiratory distress, defined by the World Health Organization as an elevated respiratory rate or low oxygen saturation, was associated with a 22% mortality rate [14]. Our group has also shown in a prospective study of the etiology of infection after chemotherapy at the Uganda Cancer Institute, that tuberculosis was responsible for 19% of infections [6]. It is possible that some of the clinical diagnoses of pneumonia in our study participants were due to tuberculosis, which has implications for patient management and infection control in this vulnerable patient population. Furthermore, patients admitted to the hospital with lower respiratory tract infection after chemotherapy are more likely to have invasive fungal infection with pulmonary aspergillosis as well as other molds; however, this is not well described in sSA [15, 16]. The myriad possibilities for infection and the lack of microbiological diagnoses in our study underscores the importance of prioritizing improved microbiological diagnostic capacity in low-resource settings.

The alarming 42% 30-day case fatality rate in patients with cancer hospitalized with suspected infection after chemotherapy at MRRH is higher than the 25% 30-day case fatality rate found in patients with postchemotherapy fever and a history of solid tumor at the Uganda Cancer Institute in Kampala [6]. Despite having relatively few participants with a hematologic cancer, the case fatality rate in our study was similar to the 46% 30-day case fatality rate in a study of high-risk patients with hematologic cancers and febrile neutropenia that also took place at the Uganda Cancer Institute [8]. The reason for the discrepancy in case fatality rates between these 2 facilities in Uganda may be explained in part by greater availability of human and material resources at the Uganda Cancer Institute than at MRRH, including routine access to broad-spectrum antibiotics and a clinical microbiology laboratory. In addition, participants in our study were more severely ill at admission than those with solid tumors at the Uganda Cancer Institute, as 24% of participants in our study had a high-risk UVA score >4 compared with only 8% in the study from the Uganda Cancer Institute [6]. The Uganda Cancer Institute is located in the capital city, Kampala, whereas MRRH is located in rural southwestern Uganda. MRRH's wide catchment area makes access to care difficult for rural dwellers, which may lead to greater severity of illness by the time of presentation to hospital.

Poor functional status, as defined by an elevated ECOG performance status scale score, was independently associated with death at 30 days in both the qSOFA and UVA score multivariable models. Elevated ECOG score may be due to advanced cancer or the adverse effects of chemotherapy or surgery [17]. In a study of noncancer patients with sepsis and a high prevalence of HIV in Uganda, the Karnofsky performance scale score, which is also commonly used to assess functional status in patients with cancer, was a significant predictor of in-hospital mortality rate and early fluid resuscitation [18]. This finding suggests that patients appearing more severely ill receive early interventions but may still die despite best efforts due to underlying advanced chronic disease. Similarly, in a study from the Netherlands, ECOG performance score was associated with in-hospital and long-term mortality rates in critically ill patients with cancer [19]. In previous studies of patients admitted with sepsis in Uganda, in-hospital mortality rates were predicted by ambulatory status and mid–upper arm circumference, which are also measures of chronic illness [20–22]. Taken together, these data suggest that death from infection in our study participants was likely due to a combination of underlying advanced chronic illness and the severity of acute illness, as determined by either qSOFA or UVA score.

Both qSOFA and UVA scores were independently associated with death at 30 days, had similar good discriminative ability, and could be used to clinically assess severity of illness in patients with cancer and infection in similar settings to MRRH. Our findings are similar to the solid tumor study from the Uganda Cancer Institute, which found that a UVA score >4 was associated with increased death at 30 days, with a hazard ratio of 14.5 [6]. In our study, there was considerable overlap between participants with clinician-diagnosed sepsis and those with s qSOFA score ≥2, a UVA score 2–4, or a UVA score >4. However, clinician-diagnosed sepsis had a relatively poor PPV (0.46) for death at 30 days. Although it was not statistically significant, we found an increase in PPV for death at 30 days for a UVA score >4 compared with a qSOFA score ≥2 (0.72 vs 0.63), suggesting that the use of qSOFA or UVA scores can augment risk stratification for patients with cancer admitted to the hospital with postchemotherapy infection. It is not clear from our study how clinicians diagnosed sepsis, but adhering to a common diagnosis of sepsis based on qSOFA or UVA scores would provide a standardized measure of expected mortality rate and assist in guiding appropriate and available resources for managing critically ill patients with postchemotherapy infection. The increased PPV for death at 30 days for the UVA score may make it preferable for guiding triage and clinical intervention decisions.

Our study had limitations. First, it was conducted at a single institution, so extrapolations to other sites should be made with caution. Second, clinical microbiology was not available in our study, so we were unable to associate mortality outcomes with microbiological diagnosis. Accordingly, we do not know whether antimicrobial therapy was appropriate for underlying infections. Studies from the Uganda Cancer Institute have documented a high prevalence of tuberculosis and multidrug-resistant bacteria, which would not be treated by ceftriaxone, the standard empiric antimicrobial therapy at MRRH [6, 23, 24]. Furthermore, we were not able to diagnose invasive fungal infections that may have been untreated. Third, our study population included participants with predominantly solid tumors, and relatively few had hematologic cancer. Due to a limited capacity for providing blood products to transfusion-dependent patients, such as those with acute leukemia, MRRH registers only leukemia patients who cannot afford to travel for treatment at the Leukemia and Lymphoma Department at the Uganda Cancer Institute in Kampala. The relative lack of participants with hematologic cancers in our study could have lowered the case fatality rate, since neutropenic fever, which is more prevalent in patients with leukemia, is associated with high case fatality rates.

Finally, autopsy was infrequent for participants who died in the hospital, and many of our study participants died after discharge. It is therefore possible that the final cause of death was due to factors other than infection. Despite these limitations, our study provides new and comprehensive insights into the case fatality rate and factors associated with death at 30 days among patients with postchemotherapy infection in southwestern Uganda. We have also shown that qSOFA and UVA scores are useful measures of severity of illness, which could be used to guide clinical management.

In conclusion, participants with postchemotherapy infection in southwestern Uganda, we found that the case fatality rate was high, with 42% succumbing by 30 days after admission to hospital. Both chronic and acute illnesses, as measured by ECOG, qSOFA, and UVA scores, were associated with mortality rates. Pneumonia was the leading cause of infection, but the inability to make a microbiological diagnosis meant that antimicrobial therapy may have been inappropriate in this immunosuppressed and vulnerable patient population. Our study provides insights to improve outcomes from postchemotherapy infection in this setting through use of standardized clinical risk score risk stratification and a focus on improving the ability to make a microbiological diagnosis. Given the lower number of liquid tumors in our participants, our findings are likely more generalizable to patients with solid tumors at the Uganda Cancer Institute and similar institutions in Eastern Africa, where cancer services may be similarly underresourced. Future studies could incorporate additional laboratory evaluation to better classify infection types and disease severity. Expanding this research to include additional similar institutions in Africa would provide a more comprehensive estimation of mortality rates. In addition, exploring potential geographic variations in infection rates and pathogens could yield insights into regional disparities.

## Supplementary Material

ofae634_Supplementary_Data

## Supplementary Data

Supplementary materials are available at *Open Forum Infectious Diseases* online. Consisting of data provided by the authors to benefit the reader, the posted materials are not copyedited and are the sole responsibility of the authors, so questions or comments should be addressed to the corresponding author.
