# Supplementary material for: Factors Associated With Death at 30 Days and Evaluation of Clinical Risk Scores Among Patients With Cancer Admitted With Postchemotherapy Infection in Uganda: A Prospective Cohort Study
Source: Open Forum Infect Dis. 2024 Oct 25;11(11):ofae634. doi: 10.1093/ofid/ofae634 (PMC11565409; doi:10.1093/ofid/ofae634)
Supplement: ofae634_Supplementary_Data [file ofae634_supplementary_data.zip › Supp.Table.1.docx]

| **Score component** | **qSOFA** | **Points** | **UVA** | **Points** |
| --- | --- | --- | --- | --- |
| Heart rate (beats/minute)* | — | — | ≥120 | 1 |
| Respiratory rate (breaths/minute) | ≥22 | 1 | ≥30 | 1 |
| Systolic blood pressure (mmHg) | ≤100 | 1 | <90 | 1 |
| Temperature (^o^C)* | — | — | <36 | 2 |
| Oxygen saturation (%)* | — | — | <92 | 2 |
| Living with HIV (yes; no or unknown)* | — | — | Yes | 2 |
| Glasgow coma scale score (n) | <15 | 1 | <15 | 4 |

**Supplementary Table 1.** Quick Sequential Organ Failure Assessment (qSOFA) and Universal Vital Assessment (UVA) score components, cut-offs, and associated points.

*Variable not included in the qSOFA score
