# Supplementary material for: Factors Associated With Death at 30 Days and Evaluation of Clinical Risk Scores Among Patients With Cancer Admitted With Postchemotherapy Infection in Uganda: A Prospective Cohort Study
Source: Open Forum Infect Dis. 2024 Oct 25;11(11):ofae634. doi: 10.1093/ofid/ofae634 (PMC11565409; doi:10.1093/ofid/ofae634)
Supplement: ofae634_Supplementary_Data [file ofae634_supplementary_data.zip › Supp.Table.2.docx]

**Supplementary Table 2.** Malignancies among cancer patients admitted with post-chemotherapy infection at Mbarara Regional Referral Hospital, Uganda, 2022-2023.

|  | **Participants (%)**  **(N=150)** |
| --- | --- |
| Esophageal cancer | 27 (18) |
| Gastric cancer | 18 (12) |
| Breast cancer | 15 (10) |
| Colorectal cancer | 13 (9) |
| Prostate cancer | 12 (8) |
| Lung cancer | 7 (5) |
| Non-Hodgkin’s lymphoma | 7 (5) |
| Chronic myelogenous leukemia | 6 (4) |
| Choriocarcinoma | 4 (3) |
| Pancreatic cancer | 4 (3) |
| Acute lymphocytic leukemia | 3 (2) |
| Chronic lymphocytic leukemia | 3 (2) |
| Kaposi sarcoma | 3 (2) |
| Nasopharyngeal carcinoma | 3 (2) |
| Osteosarcoma | 3 (2) |
| Cancer of unknown primary | 2 (1) |
| Multiple myeloma | 2 (1) |
| Ovarian cancer | 2 (1) |
| Penile cancer | 2 (1) |
| Thyroid cancer | 2 (1) |
| Other cancer* | 10 (7) |

*Other cancers included one each of the following: acute myeloid/promyelocytic leukemia, buccal squamous cell carcinoma, Castleman’s disease, cervical cancer, dermatofibrosarcoma, gastrointestinal stromal tumor, gastrointestinal junction adenocarcinoma, gestational trophoblastic neoplasia, Hodgkin’s lymphoma, and sarcoma.
