# Supplementary material for: Factors Associated With Death at 30 Days and Evaluation of Clinical Risk Scores Among Patients With Cancer Admitted With Postchemotherapy Infection in Uganda: A Prospective Cohort Study
Source: Open Forum Infect Dis. 2024 Oct 25;11(11):ofae634. doi: 10.1093/ofid/ofae634 (PMC11565409; doi:10.1093/ofid/ofae634)
Supplement: ofae634_Supplementary_Data [file ofae634_supplementary_data.zip › Supp.Table.3.docx]

**Supplementary Table 3.** Clinically diagnosed infections among cancer patients admitted with post-chemotherapy infection at Mbarara Regional Referral Hospital, Uganda, 2022-2023.

|  | **Participants (%)**  **(N=150)** |
| --- | --- |
| Pneumonia | 66 (42) |
| Sepsis | 54 (36) |
| Gastroenteritis | 22 (14) |
| Mucositis | 18 (12) |
| Abscess | 17 (11) |
| Oral thrush | 15 (10) |
| Pyelonephritis | 13 (8) |
| Central nervous system infection | 9 (6) |
| Intra-abdominal infection | 9 (6) |
| Infected tumor mass | 5 (3) |
| Prostatitis | 5 (3) |
| Cellulitis | 5 (3) |
| Peritonitis | 4 (3) |
| Esophagitis | 3 (2) |
| Neutropenic fever | 2 (1) |
| Sinusitis | 2 (1) |
| Vaginitis | 2 (1) |
| Otitis | 1 (<1) |
| Cholangitis | 1 (<1) |
| Malaria | 1 (<1) |

The percentages of diagnoses sum to greater than 100% as more than one diagnosis was possible per participant.
