# Supplementary material for: Factors Associated With Death at 30 Days and Evaluation of Clinical Risk Scores Among Patients With Cancer Admitted With Postchemotherapy Infection in Uganda: A Prospective Cohort Study
Source: Open Forum Infect Dis. 2024 Oct 25;11(11):ofae634. doi: 10.1093/ofid/ofae634 (PMC11565409; doi:10.1093/ofid/ofae634)
Supplement: ofae634_Supplementary_Data [file ofae634_supplementary_data.zip › Supp.Table.4.docx]

| **Sepsis diagnosis** | **Sensitivity**  **(95% CI)** | **Specificity**  **(95% CI)** | **PPV**  **(95% CI)** | **NPV**  **(95% CI)** |
| --- | --- | --- | --- | --- |
| Clinician-diagnosed sepsis | 0.42 (0.30-0.56) | 0.68 (0.58-0.78) | 0.46 (0.36-0.57) | 0.65 (0.58-0.70) |
| qSOFA ≥2 | 0.60 (0.47-0.72) | 0.74 (0.64-0.83) | 0.63 (0.53-0.72) | 0.72 (0.65-0.78) |
| UVA <2, low risk | 0.22 (0.13-0.34) | 0.54 (0.43-.65) | 0.26 (0.17-0.37) | 0.49 (0.43-0.55) |
| UVA 2-4, medium risk | 0.36 (0.25-0.50) | 0.57 (0.46-0.68) | 0.38 (0.29-0.48) | 0.56 (0.49-0.62) |
| UVA >4, high risk | 0.41 (0.29-0.54) | 0.89 (0.80-0.94) | 0.72 (0.58-0.83) | 0.68 (0.63-0.72) |

**Supplementary Table 4.** Comparison of performance for identifying 30-day mortality between clinician-diagnosed sepsis, and qSOFA and UVA risk score categories among cancer patients admitted with post-chemotherapy infection at Mbarara Regional Referral Hospital, Uganda, 2022-2023.

Abbreviations: NPV, negative predictive value; PPV, positive predictive value
