# Supplementary figures and images for: Factors Associated With Death at 30 Days and Evaluation of Clinical Risk Scores Among Patients With Cancer Admitted With Postchemotherapy Infection in Uganda: A Prospective Cohort Study
Source: Open Forum Infect Dis. 2024 Oct 25;11(11):ofae634. doi: 10.1093/ofid/ofae634 (PMC11565409; doi:10.1093/ofid/ofae634)

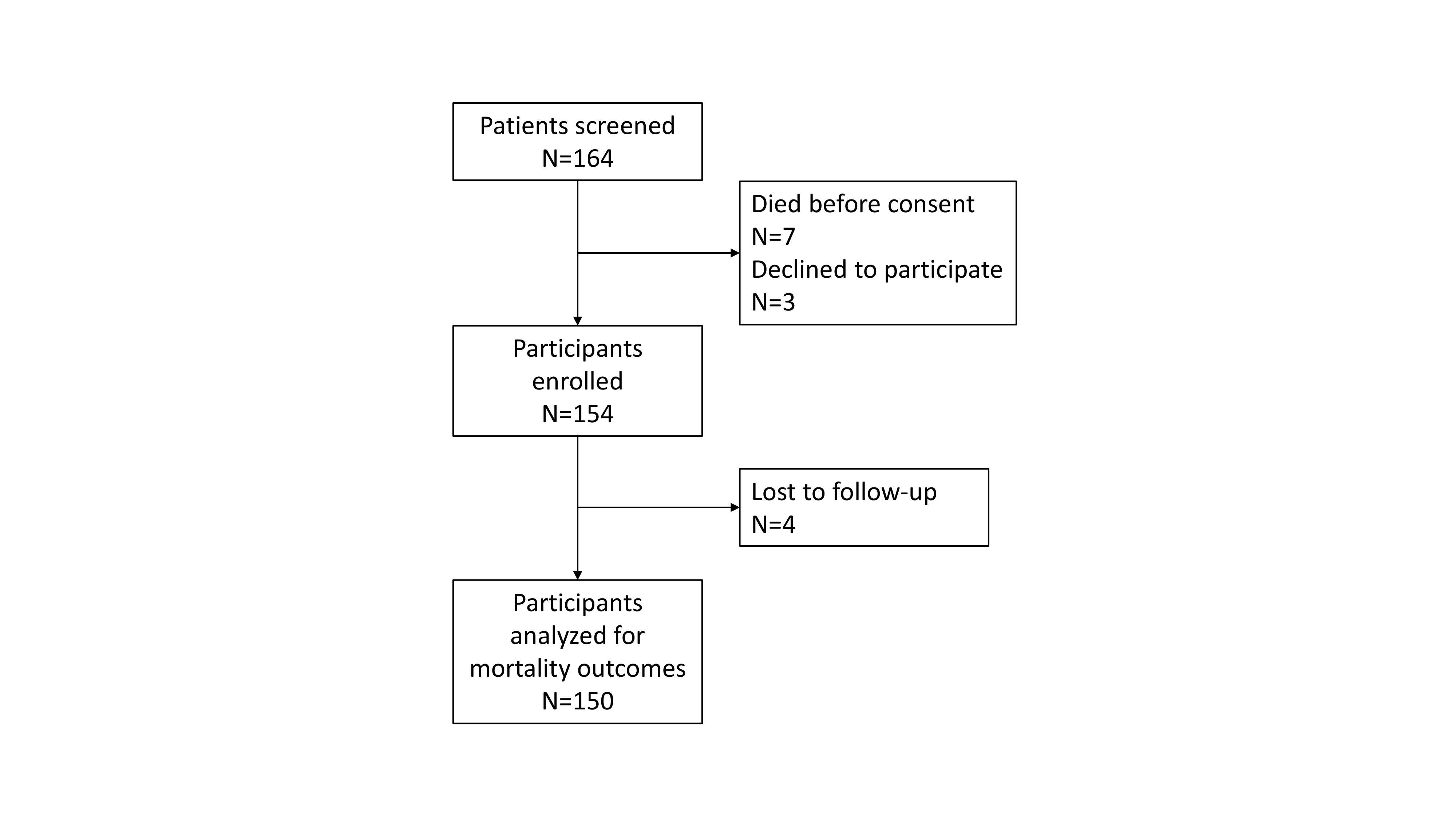

Supplement: ofae634_Supplementary_Data [file ofae634_supplementary_data.zip › Supp.Figure.1.png]
